# 1-[2-(Carboxy­meth­oxy)phen­yl]-*N*-(4-chloro­phen­yl)methanimine oxide^1^

**DOI:** 10.1107/S160053680905137X

**Published:** 2009-12-04

**Authors:** Janet M. S. Skakle, Edward R. T. Tiekink, James L. Wardell, Solange M. S. V. Wardell

**Affiliations:** a Department of Chemistry, University of Aberdeen, Old Aberdeen AB15 5NY, Scotland; b Department of Chemistry, University of Malaya, 50603 Kuala Lumpur, Malaysia; c Centro de Desenvolvimento Tecnológico em Saúde (CDTS), Fundação Oswaldo Cruz (FIOCRUZ), Casa Amarela, Campus de Manguinhos, Av. Brasil 4365, 21040-900, Rio de Janeiro, RJ, Brazil; d CHEMSOL, 1 Harcourt Road, Aberdeen AB15 5NY, Scotland

## Abstract

In the title resonance conformer, C_15_H_12_ClNO_4_, the central C–N bond [1.297 (2) Å] has considerable double-bond character and the N–O bond [1.3215 (18) Å] indicates formal negative charge on the oxygen atom. Considerable deviations from co-planarity are evident in the mol­ecule, with both benzene rings twisted out of the central C–C–N–C plane [the dihedral angle formed between the rings = 81.99 (8)°]. Similarly, the carboxylic acid residue occupies a position almost normal to the plane of the benzene ring to which it is connected [C—C—O—C torsion angle = −78.42 (17)°]. The most prominent inter­molecular inter­actions involve the carboxylic acid the N^+^–O^−^ residues with the O—H⋯O hydrogen bonds leading to helical supra­molecular chains along the *b* axis. These chains are connected into layers *via* C–H⋯O_carbon­yl_ inter­actions and the layers are consolidated into the crystal structure by C–H⋯Cl contacts.

## Related literature

For the synthesis, see: Forrester *et al.* (1974 ▶).
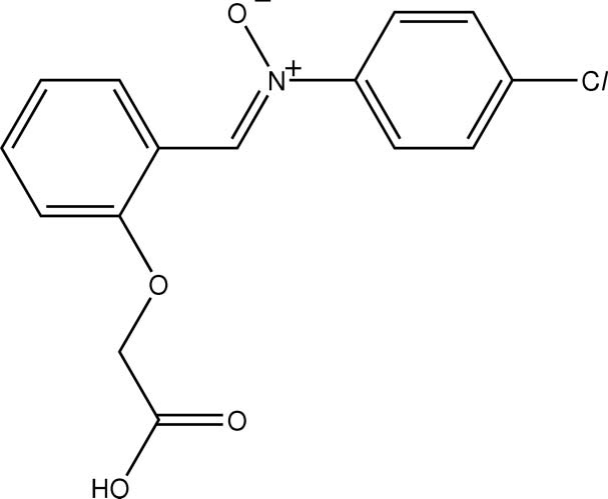


## Experimental

### 

#### Crystal data

C_15_H_12_ClNO_4_
*M* *_r_* = 305.72
Monoclinic, 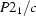

*a* = 7.6631 (2) Å
*b* = 19.3034 (5) Å
*c* = 9.6305 (3) Å
β = 107.083 (1)°
*V* = 1361.73 (7) Å^3^
*Z* = 4
Mo *K*α radiation
μ = 0.30 mm^−1^
*T* = 120 K
0.26 × 0.14 × 0.12 mm

#### Data collection

Bruker–Nonius 95mm CCD camera on κ-goniostat diffractometer
Absorption correction: multi-scan (*SADABS*; Sheldrick, 2003 ▶) *T*_min_ = 0.760, *T*_max_ = 1.000
15210 measured reflections
3113 independent reflections
2589 reflections with *I* > 2σ(*I*)
*R*_int_ = 0.043

#### Refinement

*R*[*F* ^2^ > 2σ(*F* ^2^)] = 0.038
*wR*(*F* ^2^) = 0.109
*S* = 1.10
3113 reflections
191 parameters
H-atom parameters constrained
Δρ_max_ = 0.40 e Å^−3^
Δρ_min_ = −0.46 e Å^−3^

### 

Data collection: *COLLECT* (Hooft, 1998 ▶); cell refinement: *DENZO* (Otwinowski & Minor, 1997 ▶) and *COLLECT*; data reduction: *DENZO* and *COLLECT*; program(s) used to solve structure: *SHELXS97* (Sheldrick, 2008 ▶); program(s) used to refine structure: *SHELXL97* (Sheldrick, 2008 ▶); molecular graphics: *DIAMOND* (Brandenburg, 2006 ▶); software used to prepare material for publication: *publCIF* (Westrip, 2009 ▶).

## Supplementary Material

Crystal structure: contains datablocks global, I. DOI: 10.1107/S160053680905137X/pb2014sup1.cif

Structure factors: contains datablocks I. DOI: 10.1107/S160053680905137X/pb2014Isup2.hkl

Additional supplementary materials:  crystallographic information; 3D view; checkCIF report

## Figures and Tables

**Table 1:** Hydrogen-bond geometry (Å, °)

*D*—H⋯*A*	*D*—H	H⋯*A*	*D*⋯*A*	*D*—H⋯*A*
O2—H2⋯O4^i^	0.84	1.76	2.5834 (17)	167
C2—H2a⋯O1^ii^	0.99	2.29	3.205 (2)	154
C9—H9⋯Cl1^iii^	0.95	2.71	3.5538 (16)	148

Symmetry codes: (i) 
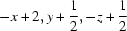
; (ii) 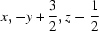; (iii) 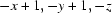.

**Fig. 1.**
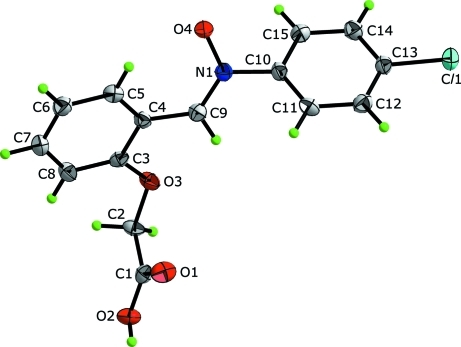
Molecular structure of (I) showing atom-labelling scheme and displacement ellipsoids at the 70% probability level.

**Fig. 2.**
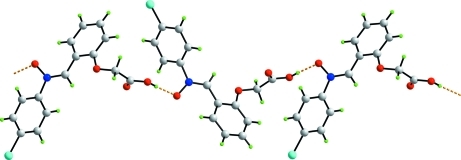
Supramolecular helical chain in (I) aligned along the b axis and mediated by O–H···O hydrogen bonds (orange dashed lines). Colour code: Cl, cyan; O, red; N, blue; C, grey; and H, green.

**Fig. 3.**
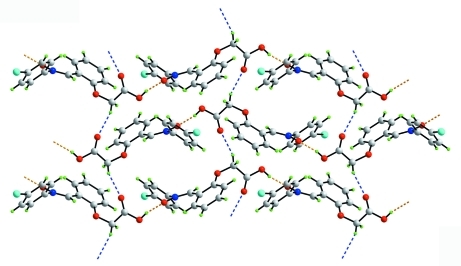
Supramolecular array in (I) in the bc plane whereby the chains illustrated in Fig. 2 are connected by C–H···O contacts (blue dashed lines). Colour code as for Fig. 2.

**Fig. 4.**
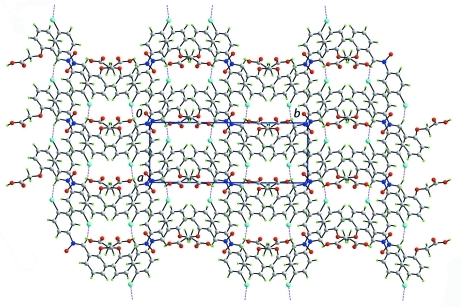
Unit-cell contents for (I) viewed in projection down the c axis showing the stacking of the layers, illustrated in Fig. 3, along the a direction with the C–H···Cl contacts shown as pink dashed lines. Colour code as for Fig. 2.

## supplementary crystallographic information

### Comment

The title compound (I) and other *N*-(2-carboxymethoxybenzylidene)aniline N-oxides are useful precursors of 4-aryl-2*H*-1,4-benzoxazin-3(4*H*)-ones on photolysis in the presence of persulfate.

The C9–N1 bond distance of 1.297 (2) Å in (I), Fig. 1, indicates significant double bond character is this bond, and the N1–O4 distance of 1.3215 (18) Å is indicative of significant negative charge on the O4 centre, indicating that (I) exists primarily as a zwitterion. The molecular structure of (I) displays considerable deviations from co-planarity of the various residues. Thus, while the central moiety is planar as seen in the C4/C9/N1/C10 torsion angle of 176.07 (14) °, both phenyl substituents are twisted out of this plane as seen in the N1/C9/C4/C3 and C9/N1/C10/C11 torsion angles of 161.63 (15) and -60.8 (2) °, respectively. The carboxylic acid residue occupies a position approximately normal to the plane of the C3—C8 phenyl ring as seen in the C1/C2/O3/C3 torsion angle of -78.42 (17) °. The most prominent hydrogen bonding interactions are of the type O–H···O and occur between the carboxylic acid-O2–H and N–O^-^ atoms. These lead to supramolecular helical chains aligned along the *b* axis, Table 1 and Fig. 2. Connections between chains are afforded by C–H···O2 interactions leading to undulating supramolecular arrays in the *bc* plane, Table 1 and Fig. 3, with the chloride atoms lying to either side. Layers stack along the *a* direction being held in place by C–H···Cl contacts, Table 1 and Fig. 4.

### Experimental

The compound was prepared according to a published procedure (Forrester *et al.*, 1974) from 2-H(O)CC_6_H_4_OCH_2_CO_2_H and 4-ClC_6_H_4_NHOH, m. pt. 484–485 K, lit. value 480–482 K. The sample used in the structure determination was grown from EtOH solution.

### Refinement

All H atoms were located from a difference map but, were geometrically placed (O–H = 0.84 Å and C–H = 0.95–0.99 Å) and refined as riding with *U_iso_*(H) = 1.2–1.5*U_eq_*(C, O).

### Crystal data

**Table:**

C_15_H_12_ClNO_4_	*F*(000) = 632
*M**_r_* = 305.72	*D*_x_ = 1.491 Mg m^−^^3^
Monoclinic, *P*2_1_/*c*	Mo *K*α radiation, λ = 0.71069 Å
Hall symbol: -P 2ybc	Cell parameters from 3158 reflections
*a* = 7.6631 (2) Å	θ = 2.9–27.5°
*b* = 19.3034 (5) Å	µ = 0.30 mm^−^^1^
*c* = 9.6305 (3) Å	*T* = 120 K
β = 107.083 (1)°	Block, colourless
*V* = 1361.73 (7) Å^3^	0.26 × 0.14 × 0.12 mm
*Z* = 4	

### Data collection

**Table:**

Bruker–Nonius 95mm CCD camera on κ-goniostat diffractometer	3113 independent reflections
Radiation source: Bruker-Nonius FR591 rotating anode	2589 reflections with *I* > 2σ(*I*)
graphite	*R*_int_ = 0.043
Detector resolution: 9.091 pixels mm^-1^	θ_max_ = 27.5°, θ_min_ = 3.1°
φ & ω scans	*h* = −9→9
Absorption correction: multi-scan (*SADABS*; Sheldrick, 2003)	*k* = −24→24
*T*_min_ = 0.760, *T*_max_ = 1.000	*l* = −12→12
15210 measured reflections	

### Refinement

**Table:**

Refinement on *F*^2^	Primary atom site location: structure-invariant direct methods
Least-squares matrix: full	Secondary atom site location: difference Fourier map
*R*[*F*^2^ > 2σ(*F*^2^)] = 0.038	Hydrogen site location: inferred from neighbouring sites
*wR*(*F*^2^) = 0.109	H-atom parameters constrained
*S* = 1.10	*w* = 1/[σ^2^(*F*_o_^2^) + (0.0552*P*)^2^ + 0.4283*P*] where *P* = (*F*_o_^2^ + 2*F*_c_^2^)/3
3113 reflections	(Δ/σ)_max_ < 0.001
191 parameters	Δρ_max_ = 0.40 e Å^−^^3^
0 restraints	Δρ_min_ = −0.46 e Å^−^^3^

### Special details

**Table:**

Geometry. All s.u.'s (except the s.u. in the dihedral angle between two l.s. planes) are estimated using the full covariance matrix. The cell s.u.'s are taken into account individually in the estimation of s.u.'s in distances, angles and torsion angles; correlations between s.u.'s in cell parameters are only used when they are defined by crystal symmetry. An approximate (isotropic) treatment of cell s.u.'s is used for estimating s.u.'s involving l.s. planes.
Refinement. Refinement of *F*^2^ against ALL reflections. The weighted *R*-factor *wR* and goodness of fit *S* are based on *F*^2^, conventional *R*-factors *R* are based on *F*, with *F* set to zero for negative *F*^2^. The threshold expression of *F*^2^ > 2σ(*F*^2^) is used only for calculating *R*-factors(gt) *etc*. and is not relevant to the choice of reflections for refinement. *R*-factors based on *F*^2^ are statistically about twice as large as those based on *F*, and *R*- factors based on ALL data will be even larger.

### Fractional atomic coordinates and isotropic or equivalent isotropic displacement parameters (Å^2^)

**Table:**

	*x*	*y*	*z*	*U*_iso_*/*U*_eq_	
Cl1	0.29830 (5)	0.38201 (2)	0.07891 (5)	0.02112 (14)	
O1	0.93820 (17)	0.79845 (6)	0.20859 (13)	0.0221 (3)	
O2	0.98035 (17)	0.87208 (6)	0.03948 (13)	0.0188 (3)	
H2	0.9383	0.9017	0.0851	0.028*	
O3	1.10293 (16)	0.69282 (6)	0.10258 (12)	0.0173 (3)	
O4	1.14983 (15)	0.47536 (6)	0.35203 (13)	0.0215 (3)	
N1	1.02235 (18)	0.51788 (7)	0.27572 (14)	0.0153 (3)	
C1	0.9942 (2)	0.81150 (8)	0.10650 (17)	0.0158 (3)	
C2	1.0864 (2)	0.75905 (8)	0.03432 (17)	0.0170 (3)	
H2A	1.0148	0.7543	−0.0691	0.020*	
H2B	1.2094	0.7762	0.0379	0.020*	
C3	1.2385 (2)	0.68188 (8)	0.23022 (17)	0.0145 (3)	
C4	1.2202 (2)	0.62054 (8)	0.30478 (17)	0.0137 (3)	
C5	1.3572 (2)	0.60303 (9)	0.43187 (18)	0.0165 (3)	
H5	1.3471	0.5614	0.4816	0.020*	
C6	1.5076 (2)	0.64566 (9)	0.48609 (19)	0.0195 (4)	
H6	1.5995	0.6337	0.5729	0.023*	
C7	1.5222 (2)	0.70602 (9)	0.41192 (19)	0.0209 (4)	
H7	1.6250	0.7353	0.4491	0.025*	
C8	1.3896 (2)	0.72460 (9)	0.28434 (19)	0.0191 (4)	
H8	1.4021	0.7660	0.2347	0.023*	
C9	1.0503 (2)	0.58210 (9)	0.24893 (17)	0.0158 (3)	
H9	0.9499	0.6065	0.1867	0.019*	
C10	0.8406 (2)	0.48799 (8)	0.21883 (17)	0.0153 (3)	
C11	0.6971 (2)	0.51583 (9)	0.25987 (18)	0.0180 (3)	
H11	0.7140	0.5563	0.3183	0.022*	
C12	0.5272 (2)	0.48347 (9)	0.21402 (18)	0.0185 (4)	
H12	0.4265	0.5014	0.2412	0.022*	
C13	0.5076 (2)	0.42485 (8)	0.12845 (17)	0.0155 (3)	
C14	0.6508 (2)	0.39793 (9)	0.08408 (18)	0.0179 (3)	
H14	0.6328	0.3585	0.0225	0.022*	
C15	0.8205 (2)	0.42961 (9)	0.13122 (18)	0.0178 (3)	
H15	0.9212	0.4117	0.1040	0.021*	

### Atomic displacement parameters (Å^2^)

**Table:**

	*U*^11^	*U*^22^	*U*^33^	*U*^12^	*U*^13^	*U*^23^
Cl1	0.0142 (2)	0.0219 (3)	0.0254 (2)	−0.00393 (15)	0.00286 (17)	−0.00054 (16)
O1	0.0261 (7)	0.0237 (7)	0.0188 (6)	0.0052 (5)	0.0103 (5)	0.0049 (5)
O2	0.0244 (6)	0.0137 (6)	0.0191 (6)	0.0040 (5)	0.0076 (5)	0.0017 (5)
O3	0.0220 (6)	0.0128 (6)	0.0153 (6)	−0.0007 (5)	0.0024 (5)	0.0011 (4)
O4	0.0154 (6)	0.0150 (6)	0.0306 (7)	0.0007 (5)	0.0015 (5)	0.0064 (5)
N1	0.0153 (6)	0.0149 (7)	0.0151 (7)	−0.0001 (5)	0.0037 (5)	−0.0002 (5)
C1	0.0129 (8)	0.0178 (9)	0.0141 (8)	0.0003 (6)	0.0000 (6)	0.0007 (6)
C2	0.0227 (8)	0.0133 (8)	0.0145 (8)	0.0019 (7)	0.0047 (7)	0.0029 (6)
C3	0.0153 (8)	0.0147 (8)	0.0142 (7)	0.0033 (6)	0.0053 (6)	−0.0014 (6)
C4	0.0150 (8)	0.0118 (8)	0.0147 (8)	−0.0007 (6)	0.0052 (6)	−0.0024 (6)
C5	0.0185 (8)	0.0146 (8)	0.0168 (8)	0.0014 (6)	0.0057 (7)	0.0004 (6)
C6	0.0164 (8)	0.0192 (9)	0.0208 (8)	0.0020 (7)	0.0021 (7)	−0.0014 (7)
C7	0.0155 (8)	0.0176 (9)	0.0277 (9)	−0.0030 (7)	0.0035 (7)	−0.0015 (7)
C8	0.0204 (8)	0.0143 (8)	0.0232 (9)	−0.0025 (7)	0.0073 (7)	0.0003 (7)
C9	0.0168 (8)	0.0153 (8)	0.0142 (8)	−0.0001 (6)	0.0029 (6)	0.0003 (6)
C10	0.0143 (8)	0.0148 (8)	0.0157 (8)	−0.0029 (6)	0.0028 (6)	0.0021 (6)
C11	0.0214 (8)	0.0146 (8)	0.0179 (8)	−0.0010 (7)	0.0060 (7)	−0.0026 (6)
C12	0.0163 (8)	0.0195 (9)	0.0207 (8)	0.0023 (7)	0.0068 (7)	0.0004 (7)
C13	0.0137 (7)	0.0158 (8)	0.0153 (8)	−0.0017 (6)	0.0015 (6)	0.0033 (6)
C14	0.0187 (8)	0.0171 (8)	0.0174 (8)	−0.0005 (7)	0.0043 (7)	−0.0022 (6)
C15	0.0169 (8)	0.0174 (9)	0.0195 (8)	0.0005 (7)	0.0060 (6)	−0.0008 (6)

### Geometric parameters (Å, °)

**Table:**

Cl1—C13	1.7419 (16)	C5—H5	0.9500
O1—C1	1.2095 (19)	C6—C7	1.388 (2)
O2—C1	1.3248 (19)	C6—H6	0.9500
O2—H2	0.8400	C7—C8	1.392 (2)
O3—C3	1.3729 (19)	C7—H7	0.9500
O3—C2	1.4260 (19)	C8—H8	0.9500
O4—N1	1.3215 (18)	C9—H9	0.9500
N1—C9	1.297 (2)	C10—C11	1.382 (2)
N1—C10	1.458 (2)	C10—C15	1.389 (2)
C1—C2	1.515 (2)	C11—C12	1.394 (2)
C2—H2A	0.9900	C11—H11	0.9500
C2—H2B	0.9900	C12—C13	1.382 (2)
C3—C8	1.392 (2)	C12—H12	0.9500
C3—C4	1.413 (2)	C13—C14	1.390 (2)
C4—C5	1.400 (2)	C14—C15	1.387 (2)
C4—C9	1.458 (2)	C14—H14	0.9500
C5—C6	1.387 (2)	C15—H15	0.9500
			
C1—O2—H2	109.5	C6—C7—H7	119.3
C3—O3—C2	119.48 (13)	C8—C7—H7	119.3
C9—N1—O4	124.48 (14)	C7—C8—C3	119.28 (16)
C9—N1—C10	119.93 (14)	C7—C8—H8	120.4
O4—N1—C10	115.58 (13)	C3—C8—H8	120.4
O1—C1—O2	125.67 (15)	N1—C9—C4	126.52 (15)
O1—C1—C2	123.94 (15)	N1—C9—H9	116.7
O2—C1—C2	110.37 (13)	C4—C9—H9	116.7
O3—C2—C1	112.21 (13)	C11—C10—C15	122.26 (15)
O3—C2—H2A	109.2	C11—C10—N1	119.43 (14)
C1—C2—H2A	109.2	C15—C10—N1	118.20 (14)
O3—C2—H2B	109.2	C10—C11—C12	118.87 (15)
C1—C2—H2B	109.2	C10—C11—H11	120.6
H2A—C2—H2B	107.9	C12—C11—H11	120.6
O3—C3—C8	124.86 (15)	C13—C12—C11	118.92 (15)
O3—C3—C4	114.96 (14)	C13—C12—H12	120.5
C8—C3—C4	120.13 (15)	C11—C12—H12	120.5
C5—C4—C3	119.07 (15)	C12—C13—C14	122.15 (15)
C5—C4—C9	123.98 (14)	C12—C13—Cl1	118.82 (12)
C3—C4—C9	116.77 (14)	C14—C13—Cl1	119.01 (13)
C6—C5—C4	120.83 (15)	C15—C14—C13	118.91 (15)
C6—C5—H5	119.6	C15—C14—H14	120.5
C4—C5—H5	119.6	C13—C14—H14	120.5
C5—C6—C7	119.21 (16)	C14—C15—C10	118.85 (15)
C5—C6—H6	120.4	C14—C15—H15	120.6
C7—C6—H6	120.4	C10—C15—H15	120.6
C6—C7—C8	121.47 (16)		
			
C3—O3—C2—C1	−78.42 (17)	C10—N1—C9—C4	176.07 (14)
O1—C1—C2—O3	−1.2 (2)	C5—C4—C9—N1	−23.3 (3)
O2—C1—C2—O3	−179.58 (13)	C3—C4—C9—N1	161.63 (15)
C2—O3—C3—C8	−15.5 (2)	C9—N1—C10—C11	−60.8 (2)
C2—O3—C3—C4	167.06 (13)	O4—N1—C10—C11	119.82 (16)
O3—C3—C4—C5	176.51 (14)	C9—N1—C10—C15	122.85 (17)
C8—C3—C4—C5	−1.0 (2)	O4—N1—C10—C15	−56.54 (19)
O3—C3—C4—C9	−8.1 (2)	C15—C10—C11—C12	1.2 (3)
C8—C3—C4—C9	174.34 (14)	N1—C10—C11—C12	−174.98 (14)
C3—C4—C5—C6	1.2 (2)	C10—C11—C12—C13	−0.3 (2)
C9—C4—C5—C6	−173.80 (15)	C11—C12—C13—C14	−1.4 (3)
C4—C5—C6—C7	−0.6 (2)	C11—C12—C13—Cl1	176.80 (12)
C5—C6—C7—C8	−0.1 (3)	C12—C13—C14—C15	2.2 (3)
C6—C7—C8—C3	0.3 (3)	Cl1—C13—C14—C15	−175.99 (12)
O3—C3—C8—C7	−176.99 (15)	C13—C14—C15—C10	−1.3 (2)
C4—C3—C8—C7	0.3 (2)	C11—C10—C15—C14	−0.4 (3)
O4—N1—C9—C4	−4.6 (2)	N1—C10—C15—C14	175.84 (14)

### Hydrogen-bond geometry (Å, °)

**Table:**

*D*—H···*A*	*D*—H	H···*A*	*D*···*A*	*D*—H···*A*
O2—H2···O4^i^	0.84	1.76	2.5834 (17)	167
C2—H2a···O1^ii^	0.99	2.29	3.205 (2)	154
C9—H9···Cl1^iii^	0.95	2.71	3.5538 (16)	148

Symmetry codes: (i) −*x*+2, *y*+1/2, −*z*+1/2; (ii) *x*, −*y*+3/2, *z*−1/2; (iii) −*x*+1, −*y*+1, −*z*.
